# The Red Cross in the Soudan

**Published:** 1898-10-29

**Authors:** 


### THE RED CROSS IN THE SOUDAN.

Sir Herbert Kitchener has not only established a claim to the gratitude of his country as a victorious general, whose success has been due to the most careful prevision of all imaginable contingencies, but he has also shown the possibility of combining, with the most skilfully arranged military operations, the maximum of care for the sick and wounded under his command. In a former campaign in Egypt, it will be remembered, the chief medical officers of the Army were deprived of the information which would have enabled them to use to the greatest advantage the limited resources under their control, and then were blamed, as most people thought very unjustly, for deficiencies which it had not been in their power to avoid. Those were the days in which the so-called "combatant" element bore unchecked sway; and in which it was difficult to make a general officer understand that organisation, in matters medical and surgical as well as in matters purely military, is the one condition essential for effective service, and that organisation could not be established without knowledge of the requirements to be provided for. With the Sirdar's force it is evident that wholly new conditions have obtained; for, from the very beginning of the expedition which led to the crowning triumph at Omdurman, the only references to the work of the medical department have been such as to show that its officers were in complete harmony with their fighting colleagues, that the sick or the wounded were receiving all possible attention, and that the labours of those by whom this attention was given were being appreciated at their true value.

In the meanwhile, we have in the newspapers of Tuesday a letter from Sir Francis Grenfell to Lord Wantage, the President of the Red Cross Society, bearing emphatic testimony to the value of the work done by the society in the campaign. The Red Cross Society is, in its essential nature, a private charity; but it had its origin in the Geneva Convention, and the first principle of its activity is that it should work in complete subordination to the military requirements of the force to which it is attached, and in strict obedience to the orders of the commanding officer. Its distinguishing badge, the red or Geneva Cross, has in time of peace been cruelly vulgarised by advertisers and amateurs, but can only be worn in actual warfare by persons who are authorised to assume it by military authority. Without regulations of this kind, strictly enforced under the sanctions of martial law, the Cross would be liable to be used as a passport by tourists, and as a disguise by spies. The society does not engage in any independent operations, but brings its help to the places where the medical officers of the army can render that help most useful to the force for which it is provided. In the recent campaign, the society furnished a hospital ship, the "May-flower," fully equipped with nurses, cots, food, medicines, and comforts, and placed under the direction of Colonel Young, as local agent, to be rendered available wherever she might be required. To this ship the medical officers of the army brought their patients, and took medical charge of them on the voyage down the Nile, the nurses of the society working under their direction, and the responsibility remaining with those upon whom it properly devolved. Similar arrangements might, it is true, have been made by the Government, but when English soldiers are fighting the battles of their country, the nation at large would not be satisfied unless it could extend to them help and comfort in somewhat more liberal measure than the strict lines of official duty would allow, and hence, we think, there is more than an excuse, there is a justification, for supplementing bare duty by voluntary effort. In this case, at least, the combination of the two has been eminently harmonious, and, according to the ungrudging testimony of Sir Francis Grenfell, eminently satisfactory and beneficial to all concerned, and an organisation which has done so much is entitled to the grateful appreciation of all Englishmen, and no small share of this appreciation will fall to the Red Cross nurses, whose good discipline, excellent conduct, and valuable services have on all sides been admitted and extolled.

